# Retrieval of a Missing Translocated Intrauterine Contraceptive Device Using Combined Laparoscopic and Cystoscopic Techniques

**DOI:** 10.1155/2024/2017479

**Published:** 2024-04-15

**Authors:** Shahd T. Natsheh, Mahmoud R. Manasra, Ahmad G. Hammouri, Nour K. Fakhorui, Yazan M. Abugharbieh, Alaa M. I. Alazzeh

**Affiliations:** ^1^Faculty of Medicine and Health Science, Palestine Polytechnic University, Hebron, State of Palestine; ^2^Department of Radiology, Al-Ahli Hospital, Hebron, State of Palestine; ^3^Department of Urology, Al-Ahli Hospital, Hebron, State of Palestine

## Abstract

**Background:**

Intrauterine contraceptive devices (IUCDs) are considered to be an effective way of preventing unwanted pregnancies. However, one significant complication associated with IUCDs is uterine perforation especially at the time of insertion and could reach the peritoneal cavity and the viscus of the adjacent organs. Intravesical migration is extremely rare. *Case Presentation*. We report a 41-year-old woman who was diagnosed with IUCD intravesical migration after she presented to our hospital complaining of persistent lower urinary tract symptoms. Laparoscopic removal was done after the failure of cystoscopic extraction.

**Conclusion:**

The IUCD must be monitored continuously by the gynaecologist, and suspicions of intravesical migration must be considered in those presenting with persistent, unexplained lower urinary tract symptoms.

## 1. Introduction

The use of intrauterine contraceptive devices (IUCDs) has been widely acknowledged as an effective nonpharmacological method for preventing unintended pregnancies [1, 2]. However, one significant complication associated with IUCDs is perforation, wherein the device moves from its original position. Migration can occur towards nearby cavities and organs surrounding the uterus, with 80% of cases involving migration into the peritoneal cavity due to uterine perforation. Nevertheless, the occurrence of intravesical migration, where the IUCD perforates both the uterus and bladder, is extremely rare [3, 4]. To date, the literature reports less than 40 cases of intravesical IUCD migration worldwide [2–8]. In this article, we present the case of a 41-year-old woman in Palestine who experienced the migration of an intrauterine contraceptive device into her urinary bladder. This case represents the first published instance of intravesical IUCD migration in Palestine. By highlighting this unique case, we aim to contribute to the existing body of knowledge surrounding IUCD complications and emphasise the importance of careful monitoring and prompt management of such occurrences. This work has been reported in line with CARE criteria, which are used by authors, journal editors, and reviewers to increase the robustness and transparency of reporting surgical cases [9].

## 2. Case Presentation

**Figure 1:**
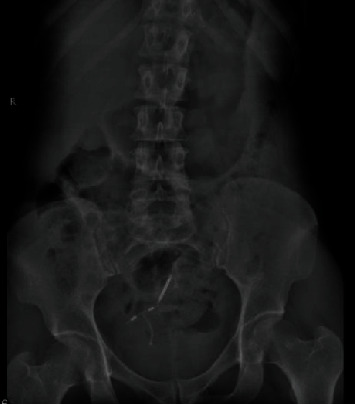
KUB shows the intrauterine device and linear radiopaque foreign material just above and medial to the IUCD in the pelvis.

**Figure 2:**
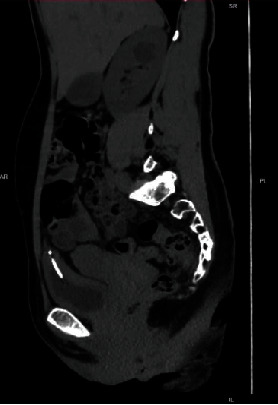
Sagittal CT: IUCD implanted within the dome of the urinary bladder.

**Figure 3:**
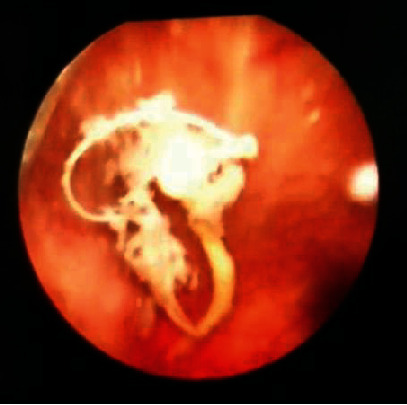
Cystoscopy showed a strip of metal substance penetrating into the posterior wall of the bladder with multiple calculi adherent to the IUCD.

A 41-year-old female G6P6 was admitted to our department with recurrent lower urinary tract symptoms for about seven months, including urinary frequency, urgency, and gross hematuria. The patient had a history of copper IUCD placement for years, after which she had an unexpected pregnancy. As no indication of a residual IUCD was found in the uterus on ultrasonography, her doctor believed that “the IUCD had spontaneously expulsed,” and a new IUCD was inserted after her last delivery 10 years ago. After that, she had no discomfort and no follow-up until seven months ago, when she experienced persistent urinary tract symptoms with a positive urine culture. Her physical examination was unremarkable. A kidney, ureter, and bladder (KUB) X-ray was done and showed an intrauterine device and linear radiopaque foreign material just above and medial to the IUCD (Figure 1), necessitating further evaluation by ultrasound, which revealed a hyperechoic shadowing material at the wall of the urinary bladder dome. The IUCD was removed by our gynaecologist, and further evaluation by computed tomography (CT) scan was done, which revealed a T-shaped foreign body (IUCD) impeded within the upper part of the posterior wall of the urinary bladder, or dome. The IUCD is noted as penetrating the bladder wall and causing local surrounding inflammation (Figure 2). The patient was diagnosed with intravesical IUCD migration and planned for cystoscopic removal of the migrated IUCD. Cystoscopy showed the stem of the IUCD penetrating the posterior wall of the urinary bladder with multiple small calculi or calcifications adherent to the intravesical portion (Figure 3). Cystoscopic removal of the migrated IUCD failed. Thus, laparoscopy was done under general anaesthesia, which revealed multiple adhesions extending from the anterior abdominal wall to the urinary bladder dome, or posterior wall. Adhenolysis was done by ligation. The IUCD arms were seen protruding out from the posterior urinary bladder wall between the serosal surface and the adhesions, with the body of the IUCD noted embedded within the urinary bladder wall. After inflating the urinary bladder with normal saline, dissection of the anterior bladder wall was done, and a cystoscope was inserted for more exploration. The IUCD was removed, and the defect was then closed by a peritoneal flap. A methylene test was done to ensure no leakage was present. The operation went smoothly with no intraoperative complications, and the patient was discharged home three days after the operation. During follow-up after two weeks, retrograde cystography revealed no contrast leakage or fistula formation, and Foley's catheter was removed.

## 3. Discussion

Intrauterine contraceptive devices (IUCDs) are widely used as reversible, effective methods of birth control. The ability to provide long-term fertility regulation makes it a preferable way and a choice for women to prevent unintended pregnancies. IUCDs work by employing diverse methods to hinder pregnancy, including the creation of an unfavourable setting for sperm or the reduction of thickness in the lining of the uterus [10].

In terms of severity, complications arising from IUCD usage encompass a range of issues. These may involve bleeding irregularities, pain, uterine perforation, vésico-uterine fistula, pelvic inflammatory disease, bowel perforation, abortion, and infection. When considering the lower urinary tract, potential complications could manifest as persistent urinary tract infections (UTIs), bladder irritation, urethral obstruction, migration of the IUCD into the lower urinary tract, and hematuria. The occurrence of IUCD migration is extremely rare, with reported rates in the literature ranging from 0.1% to 0.9% [11]. The prolonged presence of an intrauterine device (IUCD) within the bladder is considered an exceptionally uncommon event.

Anatomical and physiological considerations might have influenced this unusual migration. Anatomically, the uterus is in an anteverted and anteflexed position and is in close proximity to the bladder. This phenomenon clarifies the significant tendency for migration into the bladder [12].

Physiologically, in the time following childbirth and throughout the phase of hypoestrogenism during the breastfeeding and postpartum periods, the uterine wall undergoes a transformation, becoming notably thin and supple. This particular state of the uterine wall poses the highest likelihood for the migration of an intrauterine device (IUCD) [13].

According to literature, IUCD could migrate into the bladder through a uterovesical fistula. However, cystoscopic and laparoscopic evaluation of the urinary bladder and the uterus revealed no wall defects or fistula formation. While the exact pathophysiology of perforation remains unclear, several contributing factors have been reported to increase the risk of IUCD migration, including a prior history of caesarean section. Nonetheless, in this particular case, the patient did not have any previous caesarean sections [13, 14].

In addition, it is plausible that other variables, such as the experience and skill of the healthcare provider, could potentially influence the outcome, since theories suggest that the movement of the intrauterine contraceptive device (IUCD) into the bladder may be attributed to infection, adhesion formation, and tissue damage caused by the use of a vaginal speculum during IUCD insertion. Furthermore, if a healthcare provider encounters challenges during insertion or experiences pain or bleeding, potential acute perforation may occur [13–15]. In our case, the patient exhibited discomfort and pain without bleeding. However, it is important to note that some patients may not exhibit noticeable discomfort, and the migration of the IUCD might be discovered. Currently, there is insufficient dependable data to ascertain whether the type and composition of the intrauterine device (IUCD) have an impact on the occurrence of IUCD migration [15]. Consequently, additional research is imperative to shed light on this matter and provide more conclusive findings.

Extensive research has indicated that intrauterine contraceptive devices (IUCDs) typically exhibit adverse symptoms only in cases of perforation. Although perforation commonly occurs in the abdominal cavity or intestines, it rarely affects the urinary bladder, with studies reporting a mere 2% incidence rate. Once the IUCD breaches the bladder wall, it tends to trigger symptoms associated with bladder irritation, potentially leading to the formation of stones [15]. Urinary tract symptoms tend to dominate the clinical presentation of female patients. Our patient's symptoms align with those reported in the literature, including persistent UTI, dysuria, hematuria, and lower abdominal pain [16]. Although intravesical IUCD migration might be asymptomatic, several studies have demonstrated the necessity of IUCD removal since it exposes the patient to inflammation, as in our case.

Diagnosis might be challenging since, in some situations, the displacement of an IUCD can be overlooked during prenatal care, giving the impression that the IUCD has been expelled on its own. In these cases, healthcare providers may unknowingly insert a new IUCD. Furthermore, ultrasound examinations may concentrate on evaluating the uterus and fail to thoroughly examine the pelvic cavity, leading to missed diagnoses of ectopic IUCDs [16, 17]. Furthermore the fact that our patient did not conceive is related to a family of 6 children and probably changing domestic dynamics.

Interventions vary according to patient health status, IUCD shapes, positions, degrees of implantation within the urinary bladder wall, and the potential for calculus formation [18].

If the IUCD is partially perforated and the string is still in the vagina, attempts can be made to remove it via the vaginal route. There have been successful cases where the IUCD partially penetrated the bladder, but the strings remained in the cervix, and the IUCD was extracted through the vagina using string extraction. In addition, similar cases reported in the literature have been solved by cystoscopy in conditions where the IUCD is completely or mostly in the bladder. Besides, if part of the IUCD is intraperitoneal, it may be necessary to use laparoscopy or a combination of laparoscopy and cystoscopy [18]. However, if these methods prove challenging, open surgery becomes necessary.

According to literature that documents similar occurrences, appropriate management strategies to retrieve the IUCD are required as alternatives to cystoscopy. Three approaches to removing the device include the use of an open cystolithotomy, transurethral grasping forceps, or minimally invasive laparoscopy [5]. In our presented case, surgeons tried to extract it under cystoscopy, but it would not be expulsed, so the patient needed a laparoscopic procedure [5].

To conclude, it becomes important to consider the possibility of IUCD migration to nearby organs or cavities in cases of pregnancy with a history of IUCD placement. Patients using an intrauterine device should be advised to conduct regular device checks. If an IUCD is missed, an abdominal pelvic X-ray should be performed to rule out potential migration. Additionally, individuals with IUCDs experiencing lower urinary tract symptoms like frequent urination, urgency, and hematuria should be cautious of possible bladder involvement due to IUCD perforation. As these cases are infrequent, urologists and obstetricians must remain vigilant and well-informed about this issue.

## Additional Points

*Patient Perspective*. Thanks are due to all the medical staff who saved me from suffering for several months of pain without open surgery.
